# The prognostic role of diabetes mellitus type 2 in the setting of hepatocellular carcinoma: a systematic review and meta-analysis

**DOI:** 10.3325/cmj.2022.63.176

**Published:** 2022-04

**Authors:** Anna Mrzljak, Maja Cigrovski Berković, Francesco Giovanardi, Lai Quirino

**Affiliations:** 1 Department of Gastroenterology and Hepatology, University Hospital Center Zagreb, University of Zagreb School of Medicine, Zagreb, Croatia; 2 Department of Endocrinology, Diabetes, Metabolism and Clinical Pharmacology, University Hospital Dubrava, Zagreb, Croatia; 3 General Surgery and Organ Transplantation Unit, Department of Surgery, Sapienza University of Rome, Rome, Italy; 4 Sapienza University of Rome, Rome, Italy

## Abstract

**Aim:**

To evaluate the effect of diabetes mellitus type 2 (T2DM) on the outcomes after treatment of hepatocellular carcinoma (HCC).

**Methods:**

PubMed and Cochrane Central Register of Controlled Trials Databases were systematically searched. Three HCC clinical outcomes were explored: death, progressive disease after locoregional therapies, and recurrence. Sub-analysis was performed according to the use of potentially curative (resection, transplantation, termo-ablation) or non-curative therapies. Odds ratios (OR) and 95% confidence intervals (CI) were calculated to compare the pooled data between T2DM and non-T2DM groups.

**Results:**

A total of 27 studies were analyzed. Overall, 85.2% of articles were from Asia. T2MD was associated with an increased risk of death (OR 3.60; 95%CI 2.18-5.95; *P* < 0.001), irrespective of the treatment approach: curative (OR 1.30 95%CI 1.09-1.54; *P* = 0.003) or non-curative (OR 1.05; 95%CI 1.00-1.10; *P* = 0.045), increased HCC recurrence (OR 1.30; 95%CI 1.03-1.63; *P* = 0.03), and increased disease progressiveness (OR 1.24; 95%CI 1.09-1.41; *P* = 0.001).

**Conclusions:**

Current data provide strong evidence that T2DM unfavorably affects HCC progression and recurrence, and patients' survival after treatment, irrespective of the approach used.

The prevalence of hepatocellular carcinoma (HCC) associated with non-alcoholic fatty liver disease (NAFLD) is increasing (1,2) as the result of the globally increased prevalence of NALFD, which is estimated to be about 25% (3). NAFLD patients have a two- to three-fold increase in the risk of developing diabetes mellitus type 2 (T2DM), and the risk is even higher in those with more severe hepatic disease and fibrosis (4-6). On the other hand, patients with T2DM have a higher prevalence of non-alcoholic steatohepatitis (NASH), liver fibrosis, and end-stage liver disease (7).

Several studies have documented the relation between T2DM and the incidence of different cancer types, while the data on the relationship between T2DM and increased risk of incident HCC seem especially robust and clinically reliable (8-10). Observational studies suggest higher mortality of patients developing HCC in the presence of T2DM (11,12). On the other hand, data from meta-analyses suggest that both the risk and prognosis of patients with HCC and diabetes might be influenced by the type of anti-diabetic treatment, where metformin, unlike sulphonylurea, potentially protects against cancer and leads to better prognosis in case of cancer development (13,14).

The underlying mechanisms linking T2DM and HCC are still under scientific scrutiny. However, the interconnections between metabolic derangements characteristic for T2DM, obesity, and NAFLD suggest that insulin resistance on the hepatic and systemic level and the release of pro-inflammatory cytokines, vasoactive factors, and pro-oxidant molecules are potentially implicated in the development and progression of HCC.

With the intent to gain a better insight into this issue, we performed a meta-analysis to evaluate the effect of T2DM on poor outcomes after HCC treatment. To explore several different clinical settings, three outcomes of interest were investigated: death, progressive disease after locoregional therapies, and recurrence. Moreover, sub-analyses were performed according to the use of potentially curative (resection, transplantation, termo-ablation) or non-curative therapies.

## MATERIALS AND METHODS

### Search sources and study design

A systematic review of the published literature was undertaken focusing on the role of T2DM in the outcomes of HCC patients receiving any tumor therapy. The search strategy followed the Preferred Reporting Items for Systemic Reviews and Meta-Analysis (PRISMA) guidelines (15). In terms of inclusion criteria, data extraction, quality assessment, and statistical analysis, we used the same methodological approach as in our previous article (16).

The research question formulated in the present study included the following Patients, Intervention, Comparator, Outcome components:

Patient: patients with HCC and T2DM;

Intervention: any HCC therapy;

Comparison: patients with HCC without T2DM treated with the same approach;

Outcome: death, progressive disease, or recurrence.

We searched the PubMed and Cochrane Central Register of Controlled Trials databases using the following terms: (recurrence or death or survival) and (diabetes or T2DM) and (HCC or hepatocellular cancer or hepatocellular carcinoma or hepatoma). The search period was from 2000/01/01 to 2020/11/30.

The systematic qualitative review included only studies in English that involved human patients. Reports were excluded based on several criteria: a) data on animal models; b) studies that lacked enough clinical details; c) studies that had non-primary source data (eg, review articles, non-clinical studies, letters to the editor, expert opinions, and conference summaries). In the case of studies originating from the same center, the possible overlapping of clinical cases was examined, and the most informative study was considered eligible.

### Data extraction and definitions

After a full-text review of the eligible studies, two independent authors (QL and FG) extracted the data and crosschecked all outcomes. During article selection and data extraction, potential discrepancies were resolved by a consensus with a third reviewer (AM). Collected data included the first author of the publication, year of publication, country, and the number of treated and recurred patients according to the different therapies adopted.

### Quality assessment

Selected studies were systematically reviewed with the intent to identify potential sources of bias. The articles' quality was assessed by using the Risk of Bias In Non-randomized Studies of Interventions (ROBINS-I) tool (17).

### Statistical analysis

The results are expressed as odds ratio (OR) with 95% confidence intervals (CIs). The statistical heterogeneity was evaluated with the Higgins statistic squared (I2). I2 values of 0%-25% were considered as an index of low heterogeneity between studies, 26%-50%: moderate heterogeneity, and ≥51%: high heterogeneity. The fixed-effects model was used when low or moderate (0%-50%) heterogeneity was detected between studies, while the random effects model was used when high heterogeneity was present. The value *P* < 0.05 indicated statistical significance. The meta-analysis was performed by using OpenMetaAnalyst (http://www.cebm.brown.edu/openmeta/index.html).

## RESULTS

### Search results and study characteristics

**Figure 1:**
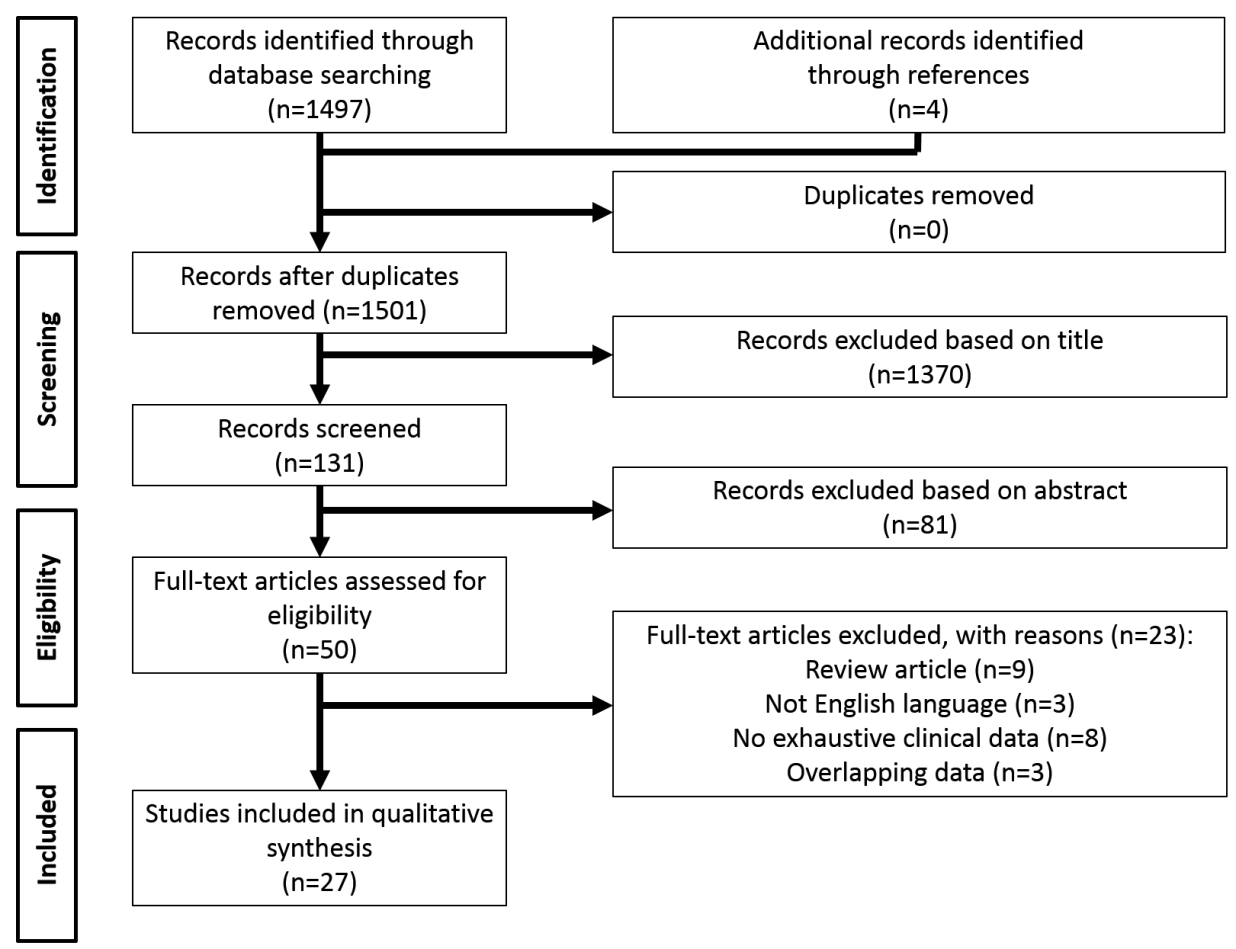
PRISMA diagram of the article selection process.

**Figure 2:**
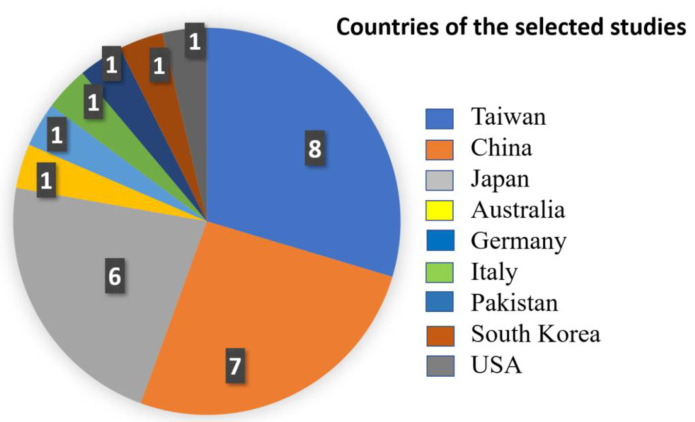
Geographical distribution of the selected studies.

The PRISMA flow diagram schematically depicts the article selection process (Figure 1). Among the 1497 articles screened, 27 were included in this review (18-44). Of these, 8 (29.6%) were published from 2001 to 2009, and 19 (70.4%) during the last decade. Twenty-three articles (85.2%) were from Asia, of which 8 (29.6%) were from Taiwan, while 2 studies (7.4%) were from Europe, 1 (3.7%) was from North America, and 1 (3.7%) from Oceania (Figure 2).

### Qualitative assessment of the included studies

All the 27 selected articles were retrospective analyses. As for the ROBINS-I tool quality assessment, all the studies had a low risk of bias.

### Review of the eligible studies

Details of the selected articles are reported in Tables 1-3. A total of 23 (85.2%) studies investigated the risk of death after any HCC treatment (Table 1) (18-27,29-37,40-42,44). Tumor recurrence was investigated in 14 (51.9%) studies (Table 2) (19,21,22,24,27,28,30,31,35,37,39-41,43), while the risk of progressive disease was reported in 7 (25.9%) studies (Table 3) (18,26,37,38,41,42,44). Overall, 5 (18.5%) studies had sample sizes of more than 1000 patients (25,34,37,41,44).

**Table 1:** Characteristics of included studies that assessed the risk of death in patients with hepatocellular carcinoma*

Ref.	First author	Year	City	Country	Study period	Design	N	Therapy	T2DM	N events	No T2DM	N events
17	Toyoda	2001	Ogaki	Japan	Jan 1990-Jun 1999	Retro	581	Mix	92	39	489	209
18	Poon	2002	Hong Kong	China	Jan 1989-Sep 1999	Retro	525	HR	62	29	463	229
19	Li	2003	Beijing	China	Jan 1998-Dec 2001	Retro	225	No surg	28	10	197	63
20	Huo	2004	Taipei	Taiwan	Apr 1996-March 2001	Retro	255	HR	41	23	214	85
							312	No surg	79	39	233	85
21	Komura	2007	Ishikawa	Japan	Jun 1987-May 2004	Retro	90	HR	30	17	60	14
22	Sumie	2007	Fukuoka	Japan	Jan 1994-Dec 2000	Retro	118	Mix	39	22	79	34
23	Kawamura	2008	Tokyo	Japan	Jan 1980-Dec 2006	Retro	40	HR/RFA	18	6	22	7
24	Huo	2009	Taipei	Taiwan	Jan 2002-Dec 2008	Retro	1713	Mix	392	235	1321	674
25	Feng	2010	Tainan City	Taiwan	Aug 2007-Jun 2008	Retro	52	TACE	14	2	38	6
26	Chen	2011	Taichung	Taiwan	Jul 2003-Jun 2009	Retro	135	RFA	53	26	82	19
28	Shau	2012	Taipei	Taiwan	Jan 2003-Dec 2004	Retro	931	Mix	185	96	746	248
29	Ting	2012	Taipei	Taiwan	Jan 2000-Dec 2008	Retro	389	HR	117	59	272	106
30	Hosokawa	2013	Tokyo	Japan	Jan 1999-Dec 2007	Retro	344	RFA	159	19	185	12
31	Bhat	2014	Rochester	USA	Jan 2005-Jun 2011	Retro	701	NA	263	170	438	257
32	Masood	2014	Lahore	Pakistan	Jun 2006-Dec 2012	Retro	282	Mix	97	50	185	95
33	Tsai	2014	Taichung	Taiwan	Jan 2000- Dec 2010	Retro	5924	HR	2962	41	2962	35
34	Wang	2014	Nanning	China	Jun 2003-Feb 2011	Retro	505	HR	134	1	371	8
35	Zhang	2014	Wuhan	China	Mar2002-Aug 2012	Retro	138	TACE	34	34	104	102
36	Chan	2016	Taipei	Taiwan	Jan 1995-Dec 2011	Retro	26 267	HR	6663	3929	19 604	10 183
							91 482	No surg	21 449	18 931	70 034	61 539
39	Yoshida	2017	Tokyo	Japan	Jan 2001-Dec 2013	Retro	224	HR	112	41	112	33
40	Li	2018	Hangzhou	China	Jan 2008-Dec 2015	Retro	11 048	LT	3136	760	7912	1730
41	Liu	2018	Beijing	China	Jan 2005-Dec 2012	Retro	308	RFA	64	34	244	172
43	Liu	2020	Shanghai	China	Apr 2011-Jan 2017	Retro	1052	TACE	289	161	763	379
**Total**	-	-	-	-	-	-	144 566	-	36 689	24 815	107 877	25 957

*Abbreviations: Ref – reference; N – number; T2DM – type 2 diabetes mellitus; retro – retrospective; HR – hepatic resection; RFA – radiofrequency ablation; TACE – trans-arterial chemo-embolization; LT – liver transplantation.

**Table 2:** Characteristics of included studies that assessed the risk of hepatocellular carcinoma recurrence*

Ref.	First author	Year	City	Country	Study period	Design	N	Therapy	T2DM	N events	No T2DM	N events
18	Poon	2002	Hong Kong	China	Jan 1989-Sep 1999	Retro	525	HR	62	35	463	275
20	Huo	2004	Taipei	Taiwan	Apr 1996-March 2001	Retro	255	HR	41	18	214	104
21	Komura	2007	Ishikawa	Japan	Jun 1987-May 2004	Retro	90	HR	30	22	60	27
23	Kawamura	2008	Tokyo	Japan	Jan 1980-Dec 2006	Retro	40	HR/RFA	18	15	22	7
26	Chen	2011	Taichung	Taiwan	Jul 2003-Jun 2009	Retro	53	RFA	53	30	82	19
27	Howell	2011	Melbourne	Australia	Jan 2000-Aug 2007	Retro	135	Mix	58	17	77	19
29	Ting	2012	Taipei	Taiwan	Jan 2000-Dec 2008	Retro	389	HR	117	63	272	116
30	Hosokawa	2013	Tokyo	Japan	Jan 1999-Dec 2007	Retro	344	RFA	159	116	185	138
34	Wang	2014	Nanning	China	Jun 2003-Feb 2011	Retro	505	HR	134	57	371	175
36	Chan	2016	Taipei	Taiwan	Jan 1995-Dec 2011	Retro	26 267	HR	6663	2851	19 604	8380
38	Choi	2017	Busan	Korea	Jan 2010-Sep 2014	Retro	58	HR	14	8	44	12
39	Yoshida	2017	Tokyo	Japan	Jan 2001-Dec 2013	Retro	224	HR	112	69	112	74
40	Li	2018	Hangzhou	China	Jan 2008-Dec 2015	Retro	11 048	LT	3136	282	7912	489
42	Billeter	2020	Heidelberg	Germany	Oct 2001-Dec 2017	Retro	88	HR	44	26	44	25
**Total**	-	-	-	-	-	-	40 021	-	10 641	3609	29 462	9860

*Abbreviations: Ref – reference; N – number; T2DM – type 2 diabetes mellitus; retro – retrospective; HR – hepatic resection; RFA – radiofrequency ablation; LT – liver transplantation.

**Table 3:** Characteristics of included studies that assessed the risk of hepatocellular carcinoma progressive disease*

Ref.	Name	Year	City	Country	Study period	Design	N	Therapy	T2DM	N events	No T2DM	N events
17	Toyoda	2001	Ogaki	Japan	Jan 1990-Jun 1999	Retro	581	Mix	92	12	489	55
25	Feng	2010	Tainan City	Taiwan	Aug 2007-Jun 2008	Retro	52	TACE	14	6	38	6
36	Chan	2016	Taipei	Taiwan	Jan 1995-Dec 2011	Retro	91 482	No surg	21 449	5314	70 033	14 685
37	Di Costanzo	2016	Naples	Italy	Oct 2008-Jun 2014	Retro	313	Sorafenib	80	9	233	41
40	Li	2018	Hangzhou	China	Jan 2008-Dec 2015	Retro	15 776	Wait list	4450	674	11 326	1557
41	Liu	2018	Beijing	China	Jan 2005-Dec 2012	Retro	308	RFA	64	54	244	169
43	Liu	2020	Shanghai	China	Apr 2011-Jan 2017	Retro	1052	TACE	289	201	763	463
**Total**	-	-	-	-	-	-	109 564	-	26 438	6270	83 126	16 976

*Abbreviations: Ref – reference; N – number; T2DM – type 2 diabetes mellitus; retro – retrospective; TACE – trans-arterial chemo-embolization; RFA – radiofrequency ablation.

### Death in HCC patients with vs without T2DM

Twenty-three studies reported post-treatment death rates in HCC patients with vs without T2DM (Table 1). Two studies contextually reported both data on post-hepatic resection and post-non- curative approach cases (21,37).

**Figure 3:**
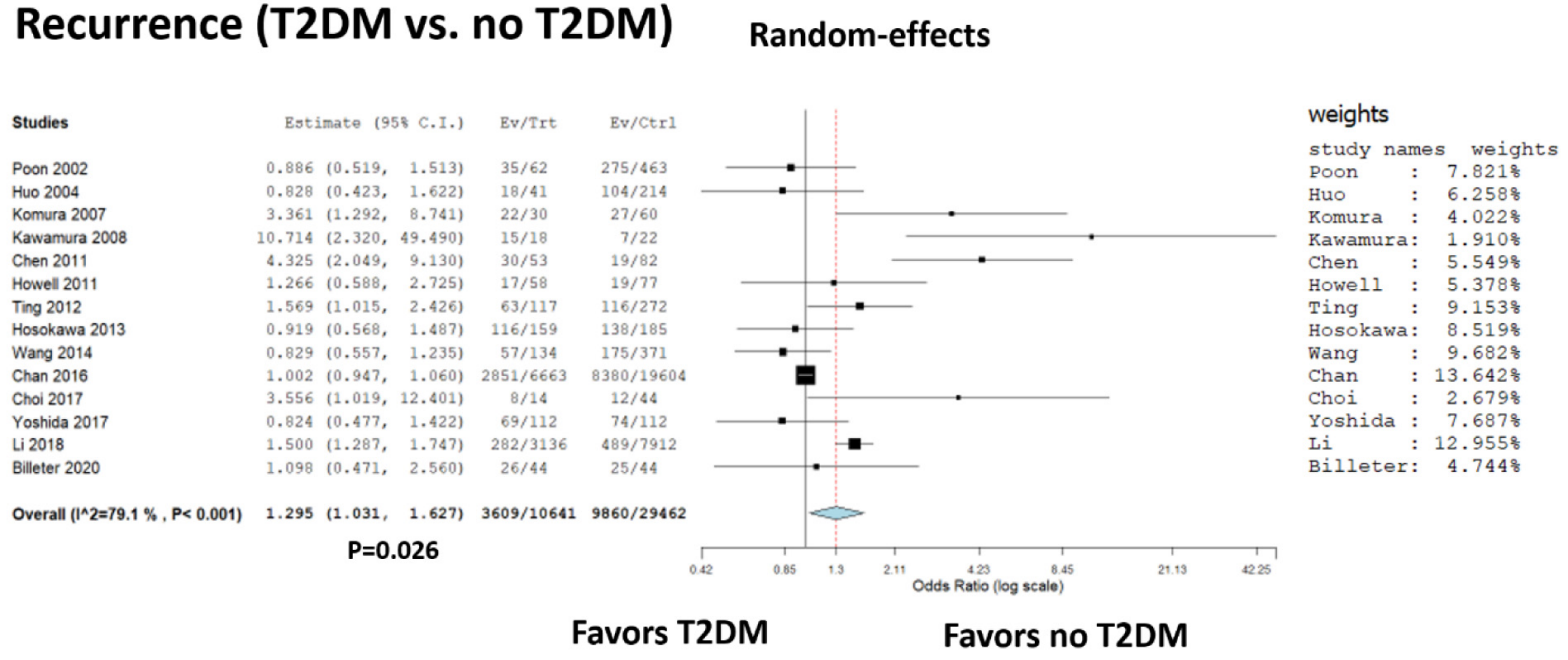
Forest plot and meta-analysis of a disease recurrence after any hepatocellular carcinoma treatment: type 2 diabetes mellitus (T2DM) vs no-T2DM patients.

A total of 144 566 patients were considered, with 50 772 (35.1%) deaths. In detail, 24 815/36 689 (67.6%) and 25 957/107 877 (24.1%) deaths were observed in the T2DM and no-T2DM group, respectively. The selected studies showed great heterogeneity, with an I2 = 99.3% (*P* < 0.001). Most of the studies showed a lower risk of death in T2DM absence (Figure 3). The summary OR showed an increased risk of death in T2DM patients, being 3.60 (95%CI 2.18-5.95; *P* < 0.001).

### Recurrence in HCC patients with vs without T2DM

**Figure 4:**
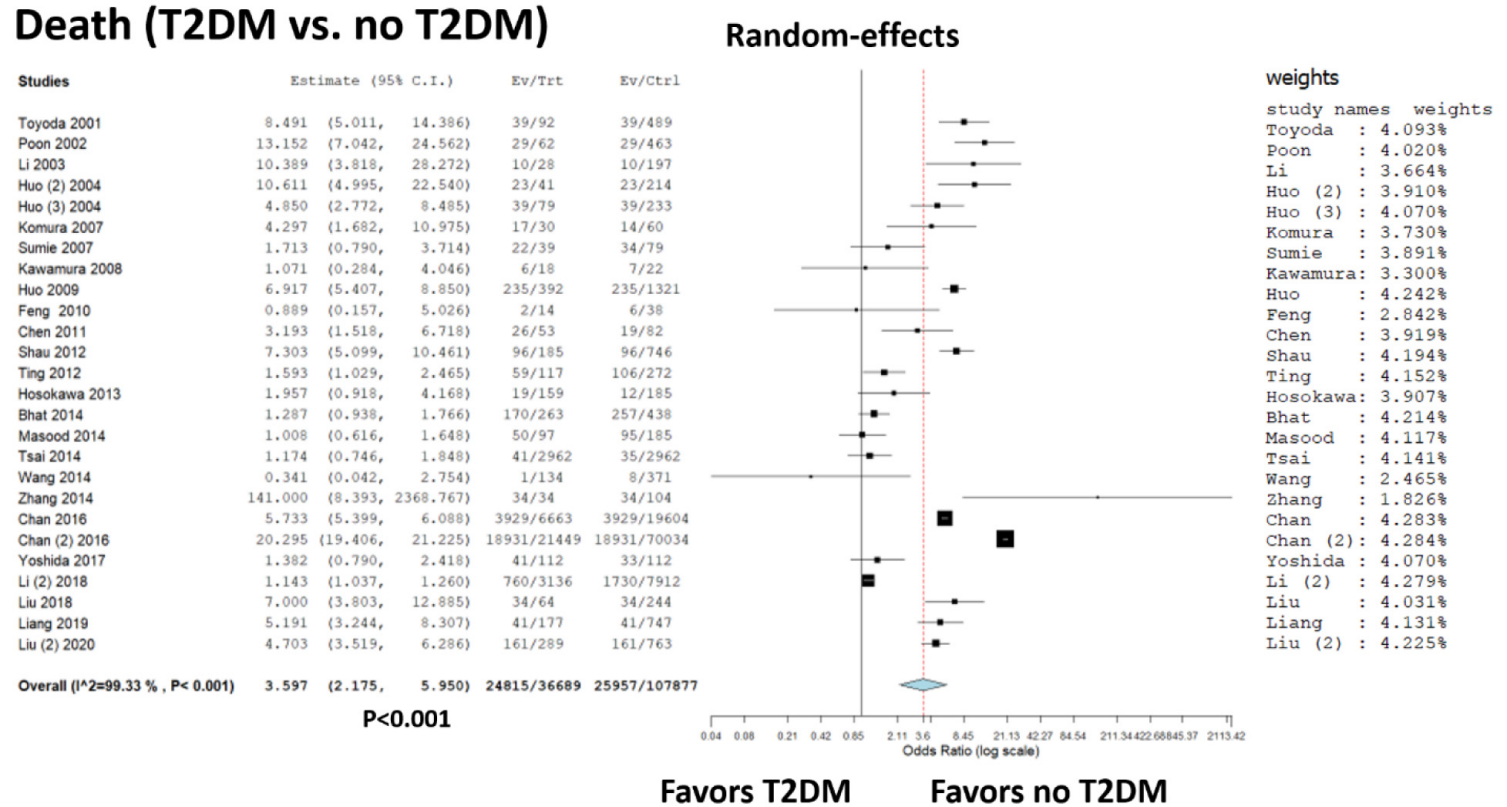
Forest plot and meta-analysis of death after any hepatocellular carcinoma treatment: type 2 diabetes mellitus (T2DM) vs no-T2DM patients.

Fourteen studies reported post-treatment recurrence rates in HCC patients with vs without T2DM (Table 2). A total of 40 021 patients were considered, with 13 469 (33.7%) recurrences. In detail, 3609/10 641 (33.9%) and 9860/29 462 (33.5%) recurrences were observed in the T2DM and no-T2DM group, respectively. The selected studies showed great heterogeneity, with an I2 = 79.1% (*P* < 0.001). Most of the studies showed a lower risk of recurrence in T2DM absence (Figure 4). The summary OR showed an increased risk of recurrence in T2DM patients, being 1.30 (95%CI 1.03-1.63; *P* = 0.03).

### Progressive disease in HCC patients with vs without T2DM

**Figure 5:**
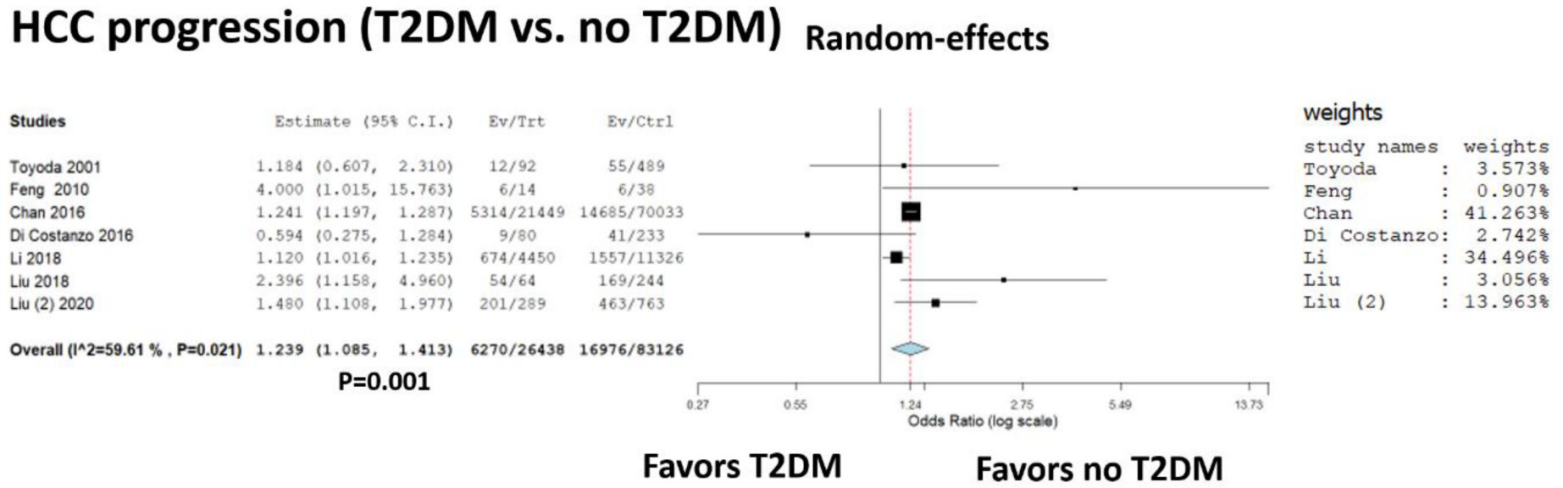
Forest plot and meta-analysis of progressive disease after any hepatocellular carcinoma treatment: type 2 diabetes mellitus (T2DM) vs no-T2DM patients.

Seven studies reported post-treatment progressive disease rates in HCC patients with vs without T2DM (Table 3). A total of 109 564 patients were considered, with 23 246 (21.2%) progressive diseases. In detail, 6270/26 438 (23.7%) and 16 979/83 126 (20.4%) progressive diseases were observed in the T2DM and no-T2DM group, respectively. The selected studies showed heterogeneity, with an I2 = 59.6% (*P* = 0.02). Most of the studies showed a lower risk of progressive disease in T2DM absence (Figure 5). The summary OR showed an increased risk of progressive disease in T2DM patients, being 1.24 (95%CI = 1.09-1.41; *P* = 0.001).

### Death in HCC patients with vs without T2DM after curative or non-curative treatments

**Figure 6:**
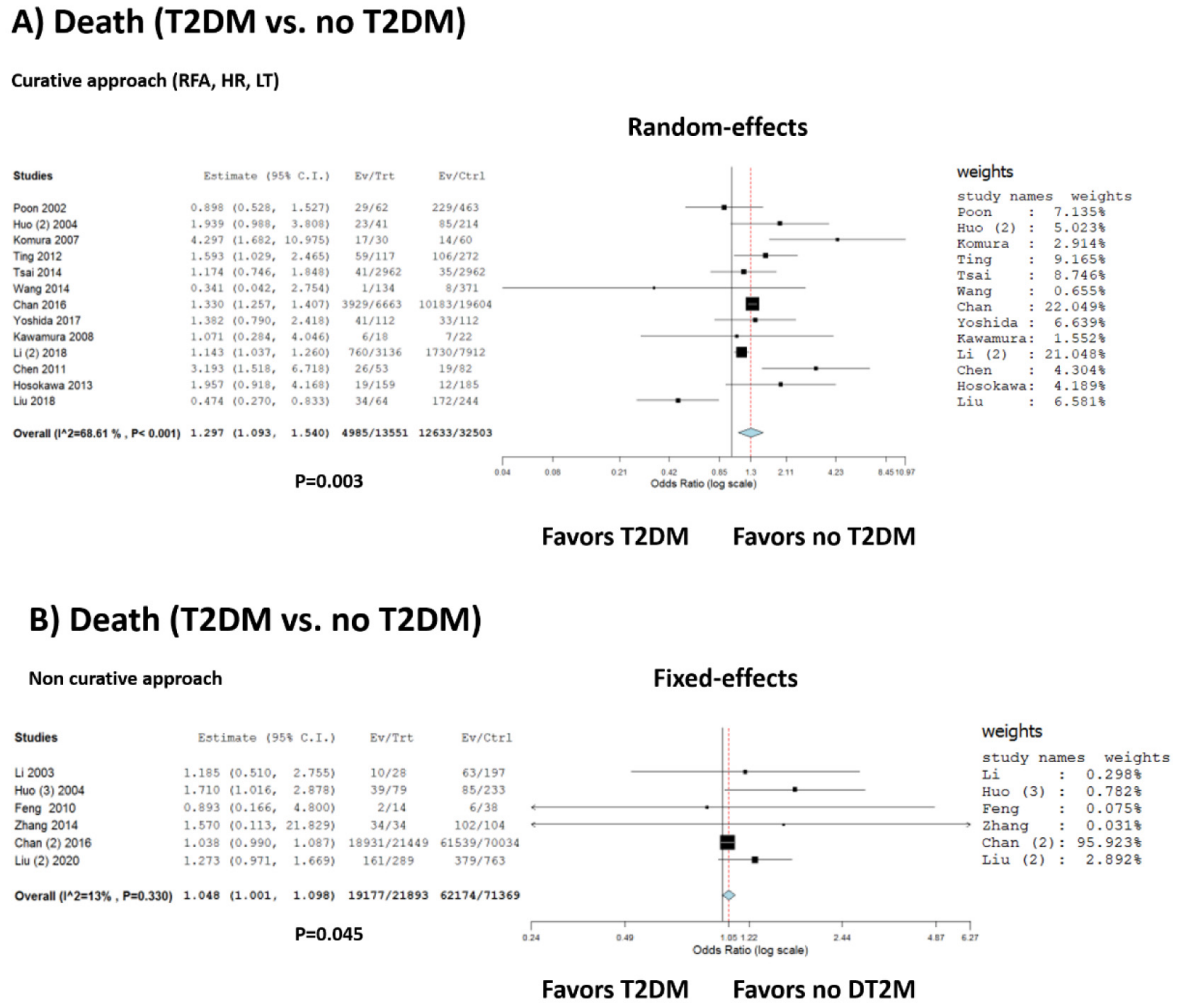
Forest plot and meta-analysis of death after (**A)** curative hepatocellular carcinoma (HCC) treatments and (**B)** non-curative HCC treatments: type 2 diabetes mellitus (T2DM) vs no-T2DM patients.

Thirteen studies reported death rates in HCC patients with vs without T2DM after a curative treatment, namely radiofrequency ablation, hepatic resection, and transplantation (Table 1). A total of 46 054 patients were considered, with 17 618 (38.3%) deaths. In detail, 4985/13 551 (36.8%) and 12 633/32 503 (38.9%) deaths were observed in the T2DM and no-T2DM group, respectively. The selected studies showed great heterogeneity, with an I2 = 68.6% (*P* < 0.001). Most of the studies showed a lower risk of death in T2DM absence (Figure 6A). The summary OR showed an increased risk of death in T2DM patients after curative approach, being 1.30 (95%CI 1.09-1.54; *P* = 0.003).

Six studies reported death rates in HCC patients with vs without T2DM after a non-curative treatment, namely any therapy other than radiofrequency ablation, hepatic resection, and transplantation. A total of 93 262 patients were considered, with 81 351 (87.2%) deaths. In detail, 19 177/21 893 (87.6%) and 62 174/71 369 (87.1%) deaths were observed in the T2DM and no-T2DM group, respectively. The selected studies showed great homogeneity, with an I2 = 13% (*P* = 0.33). Most of the studies showed a lower risk of death in T2DM absence (Figure 6B). The summary OR showed an increased risk of death in T2DM patients after non-curative approach, being 1.05 (95%CI 1.00-1.10; *P* = 0.045).

## Discussion

Our study found strong evidence that T2DM unfavorably affects HCC patients' outcomes in terms of progression and recurrence of the disease and patients' survival after treatment, irrespective of the approach used.

HCC is a common malignancy with still unfavorable prognosis (45). Among the growing risk factors for its development are metabolic derangements associated with NASH and T2DM, with insulin resistance as a common denominator (46). In addition, NAFLD-related HCC occurs both in tandem and in the absence of underlying cirrhosis, substantiating NAFLD’s tumorigenic effects (47,48). According to epidemiologic studies, T2DM can increase the incidence of HCC and unfavorably alter the prognosis of patients with HCC (49).

In reference to its multisystemic effects, T2DM, in some centers, is considered a relative or absolute contraindication for curative treatments such as liver transplantation due to poor outcomes and higher incidence of complications (50). Nevertheless, a recent meta-analysis showed that NAFLD-HCC patients could enjoy long-term survival benefits when treated with aggressive curative approaches (resection, transplantation, or thermo-ablation) with no difference in overall survival compared with non-NAFLD HCC patients (51). On the other hand, this meta-analysis provided solid evidence that T2DM patients compared with non-T2DM patients have an increased risk of death after curative HCC treatments, irrespective of the approach. Most out of 13 studies included in this meta-analysis showed a lower risk of death in T2DM absence. Furthermore, we demonstrated that the same T2DM impact on survival could also be expected after non-curative HCC approaches.

Most studies (85.2%) included in this meta-analysis were from Asia, where hepatitis B infection is still the leading cause of HCC. The incidence of alcohol- and HCV-related HCC in Asian countries is relatively stable and low, while NASH has become a growing epidemic with the prevalence of approximately 10%-20% (52,53). NASH is mainly accompanied by T2DM, dyslipidemia, and obesity, common factors leading to cardiovascular events. NASH is the fastest-growing cause of HCC in US liver transplant candidates (54), and the same is expected to occur in Asia due to changes in eating habits and sedentary lifestyles. NASH is strongly associated with HCC and liver-related mortality, yet the death in NAFLD is due mainly to cardiovascular diseases (55,56).

In addition, this meta-analysis shows that diabetes is inevitably associated with a greater risk of HCC progression. Only a study by Di Costanzo et al (38) showed longer time-to-progression (10 months vs 9 months) in 80 diabetic HCC patients treated with sorafenib compared with 233 non-diabetic HCC patients (38). Other six studies reporting data on HCC progression (17,25,36,40,41,43) showed an increased diabetes-associated risk (26 358 patients with vs 82 893 without diabetes) on HCC progression.

Overall, 14 included studies reported data on HCC recurrence (19,21,22,24,27,28,30,31,35,37,39-41,43). Robust evidence confirmed that diabetes increased HCC recurrence risk, irrespective of treatment. In the case of 508 out of 10 641 diabetic HCC patients included in the analysis, the risk of recurrence did not differ from that observed for non-diabetic HCC patients (19,21,31,35,40). Of note, the cause of HCC in the mentioned studies was mostly hepatitis virus-related.

In light of the observed results, it would be prudent to improve the prevention of T2DM by promoting a healthy diet and incorporating structured physical activity into everyday life (57). Moreover, an important role in the prevention of HCC can be played by interventions that lead to T2DM remission, such as those with glucagon-like peptide-1 receptor agonists (liraglutide and semaglutide) and bariatric surgery, which significantly reduce (>10%) and help maintain weight (58-61). The potential chemo-preventive effect of antidiabetic drugs such as metformin in HCC patients requires further clarification (62).

The present study has some limitations. First, a large part of the investigated population is from Asian countries. Therefore, the reported results should present some geographical peculiarities precluding their generalizability in Western populations. Further larger studies coming from North America and Europe are required. Another limitation relates to the great heterogeneity reported among different studies. Unfortunately, different aims and different approaches were reported in the examined studies, increasing the difficulty to obtain definitive results. We tried to minimize the heterogeneous effect by performing sub-analyses focused on specific classes of patients (ie, treated with curative vs non-curative therapies).

Further studies are required to better clarify the effect of T2DM in larger Western series and investigate the effect of anti-diabetic drugs in protecting against the risk of tumor evolution in HCC patients receiving curative or non-curative therapies.
